# Assessment of Dynamic Surface Leaching of Monolithic Polymer Mortars Comprised of Wastes

**DOI:** 10.3390/ma16062150

**Published:** 2023-03-07

**Authors:** Walid Maherzi, Ilyas Ennahal, Fatima Zahra Bouaich, Mahfoud Benzerzour, Zakia Rais, Yannick Mamindy-Pajany, Nor-Edine Abriak

**Affiliations:** 1 Laboratoire de Génie Civil et Géo-Environnement, University of Lille, IMT Nord Europe, ULR 4515—LGCgE, 59000 Lille, France; 2 Faculty of Science Dhar El Mahraz, University Sidi Mohamed Ben Abdellah, Mohammedia 28810, Morocco

**Keywords:** dynamic monolithic leaching test, polymer mortars, dredged sediments, trace elements, construction products, epoxy-resin

## Abstract

Today, the reuse of waste in building materials occupies an important place in the approach to the circularity of materials. National and European environmental regulations require ensuring the environmental safety of material-incorporating waste. For this, there are specific tests to verify that there is no health risk when using these materials. Concretely, to check the environmental acceptability of construction materials, including wastes, the release of hazardous substances into water must be assessed. In this research, we performed a diffusion test with the sequential renewal of water during a 64-day period according to the NF EN 15863 specifications on polymer mortar monoliths, common construction products used in floor-covering applications and incorporating sediments. Polymer mortars were prepared at a laboratory scale by incorporating 30 or 50% of polluted sediment for various polymer concentrations (12, 14, 16, 18, 20 and 25%). It was shown that the release of inorganic substances is limited in these hydrodynamic conditions. Among trace elements, As, Cd, Cr, Ni, Pb and Zn are lower than quantification limits in most leachates, whereas Ba, Co, Cu and V are systematically quantified at low concentration levels. This is particularly true for samples displaying the highest polymer concentration (25%) and the lowest sediment incorporation rate (30%). This is because of the low water absorption level and low porosity of polymer mortar matrices. No adverse effect is to be expected for environmental health from the leachates of these construction materials, including waterways sediments, because all the measured parameters were below the Soil Quality Decree limits applied in the Netherlands for environmental assessment of construction products.

## 1. Introduction

Today, the environmental issues related to the management of natural resources are currently considered a significant priority in nature (COP). Indeed, the need to find alternative solutions, particularly by implementing circular economy concepts and adopting new economic models, is more than necessary. In this perspective, the substitution of natural materials for alternative materials presents itself as an interesting solution vis-à-vis this socio-economic and environmental challenge, which is fully in line with a sustainable development and circular economy approach. According to this vision, numerous investigations have highlighted that dredged sediments may be reused as major or minor components in the construction industry sector. For example, in the research carried out by [1], the sediments were incorporated into the brick-manufacturing process instead of quartz sand. In addition, it has been shown that a 50% replacement of natural brick-making clay by sediments allows for reaching the compressive strength required for the American Society for Testing and Materials (ASTM) standards [2,3]. The feasibility of using dredged sediments as a partial replacement for cement in mortars was assessed by several authors [4,5]. Benslafa et al. [4] studied the variation of compressive strength at varying sediment incorporation rates (5, 10, 15 and 20% by mass of cement). The results highlighted that sediments can most suitably be substituted for 5% of the cement used. Lightweight aggregates manufactured from dredged sediments have been studied in many research works [6,7,8,9,10], and the results have shown their suitability for large-scale production due to their availability, homogeneity, mineralogical and chemical composition.

In the SEDIMATERIAUX regional framework launched in France in 2009, several innovative ways for recycling non-submersible sediments have been studied by the port of Dunkirk: landscape remodeling, use in road building as well as manufacturing of mortar blocks [11]. Today, 150,000 cubic meters have been reused in the port’s territory in the form of landscape remodeling. This landscape remodeling is designed to promote the development of biodiversity in an area of low species richness. In May 2012, the first port road was built by using dredged sediments and natural aggregates. By the end of 2013, concrete blocks, including dredged sediments, were made and used in the port’s territory to strengthen defenses against the sea. Currently, the port is studying the feasibility of using sediments to produce artificial aggregates that it will use to strengthen the coastline, which is subject to erosion. In this way, the authority port hopes to identify several treatment ways for recycling non-submersible dredged materials. More recently, the industrial research project entitled «SEDIPLAST» was launched (2015–2018) in France within the SEDIMATERIAUX framework to assess the feasibility of reusing waterways and harbor sediments in thermosetting and/or thermoplastic matrices in order to manufacture polymer mortars which could be used for floor covering applications. Technical investigations have shown that sediments can be incorporated as major component of composite products and by replacement of natural aggregates with a substitution rate of 50% [12,13]. Composite products were evaluated by mechanical, thermal and chemical tests according to UPEC specifications to validate their technical use as construction products in floor-covering applications [14].

According to the European regulation, it is commonly accepted that the use requirements of construction materials must include proof they will not have adverse effects on human health and the environment. It is expected that expected pollutant emissions in soil and water need to be quantified during the service life of construction products. Laboratory test procedures to determine the amount of substances released from construction products were established by the CEN TC 351 “construction products: assessment of release of dangerous substances”. One of the tests—CEN/TS 16637-2:2014—was developed to investigate leaching from monolithic construction products. The dynamic surface-leaching test (DSLT) intends to describe diffusion-controlled leaching processes. However, the test results cannot be used directly to derive expected environmental concentrations.

Concepts for transferring results obtained under laboratory exposure conditions to service-life conditions still need to be developed or refined [15,16]. Otherwise, the test indicates whether target substances can be leached from investigated construction products. It is also possible to compare the leachability of the target substances from different construction products according to regulatory levels from the Netherlands (Soil Quality Decree, 2008) or Germany. These tests have often been used to characterize cementitious materials [17,18]. There is very little work on the characterization of waste-based material monoliths. Often, the works carried out on the valorization of waste in the construction materials are based on leaching tests of the crushed fraction (0–4 mm) [19].

The present study is the first to assess the leaching of soluble inorganic substances from polymer mortars based on waterways sediments according to the specifications of NF EN 15863 standard. Indeed, mineral fillers (limestone and/or sands), usually used for the formulation of polymer mortars, have been partially replaced by waste that is the dredged sediment. We investigated the dynamic leaching behavior of several polymer mortar samples, including various epoxy resin rates; (12, 14, 16, 18, 20, and 25%) and two dredged sediment-incorporation rates (30 and 50% in mass) [12,19]. The objective was to identify the mechanisms for the release of chemical substances from monolith samples into water. The results of this work will subsequently make it possible to establish an environmental impact study and a life-cycle analysis of this type of material-incorporating waste.

## 2. Materials and Methods

### 2.1. Epoxy-Resin Properties

The RECKLI Epoxi EP binder used in this research study is a two-component, solvent-free, transparent epoxy-based castable resin, which was supplied by SOCECO RECKLI. Two different hardeners are proposed, and the final results are the same, but the reaction rate is changed. The hardener was selected with a reaction rate of between 40 min and 50 min. RECKLI Epoxi EP resin can be mixed with different fillers and allowed, according to the proportion of resin, to obtain mortars. Table 1 reports the main characteristics of the used epoxy resin.

### 2.2. Mineral Charges Characterization

**Figure 1:**
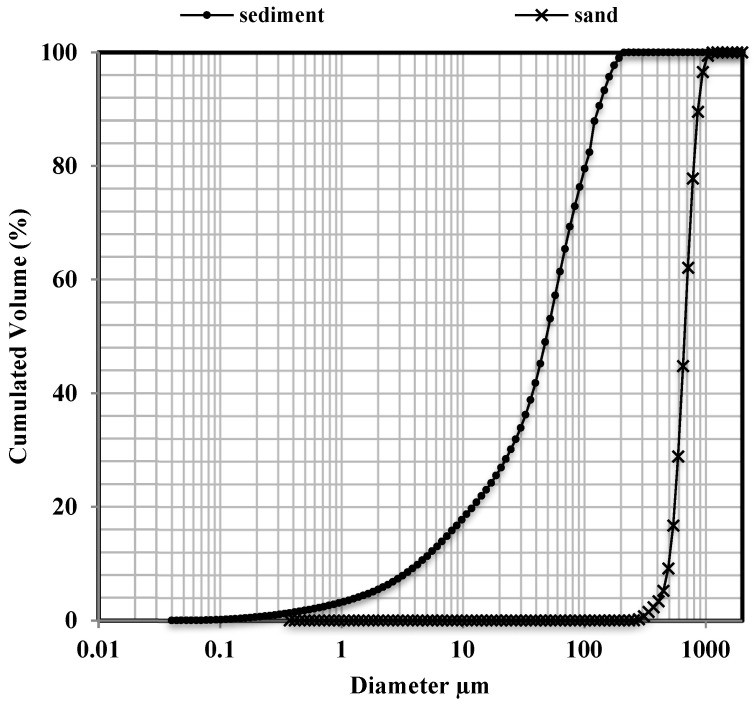
Comparison of particle size distribution in the sediment sample and the normalized sand used in the polymer mortar mixtures.

In this work, a sediment sample was used in polymer mortars; it was collected in the port of Dunkirk in the Hauts-de-France region of France. The physical parameters measured on this sediment sample are reported in Table 2. The density was measured using a Micrometrics Accupycs 1330 helium pycnometer model. This test was performed in accordance with European standard NF EN 1097-7: (2008). In accordance with standard NF EN ISO 18757: (2003), the BET surface area was also measured, thus enabling the fineness of the materials to be evaluated using a Micromeritics Autopore IV 9505 instrument. The evaluation of the organic material content is carried out by the fire loss test according to the standard XP P94-0447: (1998) consisting of calcination at 450 °C for 3 h and a measurement of the loss of mass. The methylene blue (VBS) absorption test for the evaluation of the clay was also carried out in accordance with standard NF P 94-068: (1998). Determination of the particle size was performed with an LS 13320 laser apparatus. The particle size distribution of the sediment sample is compared to the sand particles in Figure 1. The sand used in polymer mortar manufacturing is standardized sand (ISO 679 standardized sand) containing natural siliceous sand, especially in the finer fractions; the density of the sand is 2650 Kg/m^3^. The mineralogical characterizations of the sediment and sand were carried out essentially by X-ray diffraction analysis with a D2 PHASER-BRUKER6 Diffractometer with CuKα radiation, and the diffractograms are acquired at the angle 2θ = 10–80° to identify the mineralogical phases. The results indicate that the sediment consists mainly of quartz (SiO_2_) with a low presence of calcite (CaCO_3_). We also note the presence of minor mineral phases such as albite (NaAlSi_3_O_8_), orthoclase (KAlSi _3_O_8_) and muscovite (KAl_2_ (AlSi_3_O_10_)(OHF)_2_). The sand consists exclusively of crystallized silica (Quartz). Table 3 shows the elemental X fluorescence composition of the sediment. Mainly the sediment contains silicon (Si) and calcium (Ca). Iron (Fe) and aluminum (Al) are present in significant amounts.

### 2.3. Polymer Mortars Mix Design

Binder manufacturing involves mixing the base product and the hardener in a bucket for 3 min until a uniform mixture is obtained. In another bucket, the charges were mixed (sand and sediment), and then the charges were added and mixed with the binder in 2 parts, and each part was mixed for 6 min. The molds are filled in two layers; each layer is compacted using the impact table (60 shots). The polymer mortar samples were demolded after 24 h and cured in the air at 25 °C and 48% relative humidity. The composition of the mixtures and the main physical and mechanical characteristics of the mortars are described in Table 4. Porosity is a very important indicator of durability and is a key factor in interactions with the external environment in terms of permeability. Therefore, it is important to measure pore size distribution for small dry mortar fragments, according to a Micromeritics Autopore V 9600 in accordance with ISO 15901-1:2016.

It is noted that the mercury porosity is related to the quantity of aggregates and the resin-based binder. Indeed, when the mixture resin rate increases, the porosity and the water absorption decrease. Indeed, increasing the resin content makes it possible to fill the intragranular porosity and increase the density of the material. In other words, the packing density of the granular skeleton is increased by the addition of resin [13]. The presence of sediments in the polymer mortars influences the final porosity. Values measured in the formulations with 30% sediment are systematically lower than those of the formulations, including 50% of sediments in their matrices. This can be explained by the decrease in the packing density of the granular skeleton formed by the sand and the sediments when we increase the rate of sediment increases in the mixtures [13,14].

### 2.4. The Batch Leaching Test NF EN 12457-2 on Granular Constituents of Polymer Mortars

The sediment and the sand provide chemical substances in the polymer mortars, which could be leached into water. The batch leaching tests were carried out in accordance with the European standard EN 12457-2. The principle of the test consisted of exposing the crushed material to a liquid for 24 h, then analyzing the obtained eluate. Each material having a particle size less than or equal to 4 mm was tested in triplicate reduced to a maximum particle size of 4 mm. It applies to fragmented waste and sludge with a particle size less than 4 mm, with the reduction of the size of the particles meeting this criterion being possible. A test portion corresponding to 90 g (±5 g) of dry mass is placed in a one-liter flask. The material of the flask is chosen so as to limit as much as possible the interactions with the waste tested and as a function of the substances assayed during the analysis of the eluate (in our case, it is high-density polyethylene). The lixiviate used is ultra-pure water. The amount of liquid to be added is determined so that the liquid/solid ratio (L/S in L/kg of dry matter) is 10 (±2%). The flask is then shaken with a rotary shaker at 10 rpm for 24 h (±30 min). At the end of the test, the separation of the eluate from the solid is done in two steps. First, the mixture is allowed to settle for 15 min ±5 min, and then the eluate is filtered through a 0.45 μm cellulose acetate membrane. A centrifugation step can be added in case of problems. For each eluate, the pH, conductivity and temperature are systematically measured.

### 2.5. Dynamic Surface Leaching Test on Polymer Mortar Samples

**Figure 2:**
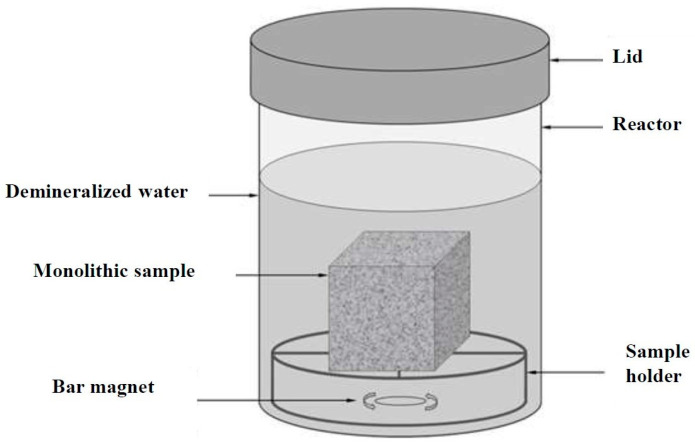
Experimental design for dynamic monolith leaching test applied on polymer mortar samples.

Leaching tests on mortar monoliths were conducted according to the EN 15863 standard with periodical chant renewal. Each monolith was placed in a plastic reactor, and a given volume of deionized water was introduced to submerge the monolith completely and reach a “volume to surface ratio” (L/A ratio) of 8 cm^3^/cm^2^ (Figure 2). The top surface of the monolith was kept at least 2 cm below the surface of the water, and the distance between the surfaces of the monolith and the walls of the reactor was kept above 2 cm. At time intervals of 0.08, 1, 2.25, 8, 14, 15, 28 and 36 days, the aqueous solution was completely removed from the reactor and replaced with the same volume of deionized water. The pH and electrical conductivity were measured immediately. Then the leachate was filtered through a cellulose-acetate membrane of 0.45 µm pore size, and the solution was analyzed within 24 h for a number of traces (As, Ba, Cd, Cr, Cu, Co, Mo, Ni, Pb, Sb, Se and Zn) major elements (Ca, Fe, K and Mg) and anions (sulfates, chlorides and fluorides), respectively, by ICP-OES and ionic chromatography.

## 3. Results and Discussion

### 3.1. Batch Leaching Test Results on Granular Constituents

Leaching parameters are reported in Table 5 for both granular constituents used in the formulations of polymer mortar. For the sand, all inorganic substances are lower than the quantification limits, except for Ba, which is released at 0.03 mg/kg of dried mass. This level is very low compared to the regulatory levels established for inert and non-inert waste storage in Europe. The pH level of around 9 suggested that the carbonate minerals detected in the sand are soluble and control the final pH in leachates. This could be explained by the measured soluble fraction measured in this batch leaching test (398 mg/kg), which remained under the regulatory levels for inert and non-inert waste storage. Inversely, the sediment released more chemical substances, but their concentration levels were relatively low for most parameters. All detected parameters (Ba, Cr, Cu, Mo, Ni, Pb, Sb, Se, Zn, chlorides, fluorides, and sulfates) are lower than the regulatory level for inert waste storage, except for Sb, which exceeded this level. It can be noted that the soluble fraction and conductivity are higher in the sediment leachate than in the sand one. This can be explained by the large diversity of minerals detected in the sediment that can be dissolved in leachates. The pH level seems to be controlled by the carbonate minerals. Finally, the sediment is considered a non-inert material on this basis. Inert materials are generally the most difficult to reuse because environmental criteria for the beneficial reuse of waste in civil engineering are often based on inert waste storage referential. However, their use remains possible in some applications as construction products.

### 3.2. Dynamic Monolith Leaching Tests

#### 3.2.1. Physicochemical Parameters: pH, Conductivity and Redox Potential

Relatively stable pH values are observed in the first three samples for the different formulations regardless of the rate of sediment incorporated. There is less variability in formulations with 50% sediment (8.5–9). In formulations containing 30% of sediments, this variability is greater, with the pH values varying between 8 and 9.4. Given the natural pH values measured on the raw constituents in previous leaching tests (sand and sediment), it appears that these formulated materials have a buffering capacity resulting from their mineralogical composition.

**Figure 3:**
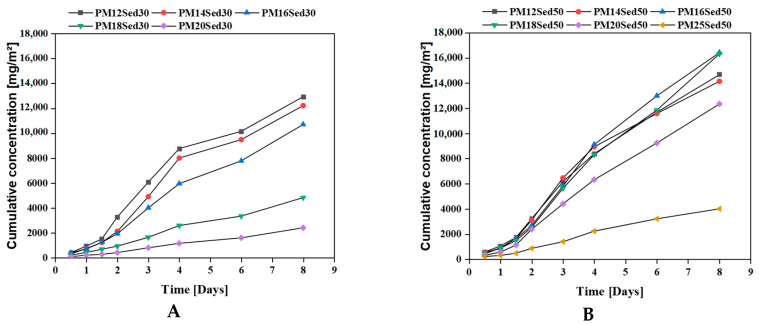
Evolution of cumulative concentration of calcium in leachates from monolithic polymer mortars containing 30% (**A**) and (**B**) 50% of sediments in their matrices.

The carbonates present in the sediment explain the good buffering capacity of the formulations, having an incorporation rate of 50%. The sand has traces of carbonates and participates indirectly in maintaining the pH. From the 4th renewal, it can be noted the drop in pH (between 1 and 2 points) to the value of 7 for the different formulations, regardless of the sediment content. The nature of the dynamic monolith leaching test makes the renewal of the solution allows the dissolution of the stock of carbonate minerals; the presence of calcium in the leachates confirms this mechanism (Figure 3). It can be noted that calcium diffusion in leachates is strongly correlated to the resin concentration in the polymer mortar. The increase in resin concentration reduces the diffusion of calcium in leachates and, therefore, carbonate dissolution. On the last three renewals, the pH remains more or stable at a neutral value in the different formulations in the presence of 30 or 50% of sediments, suggesting that the resin composition (mainly alcohol groups) plays an important role in the pH values.

**Figure 4:**
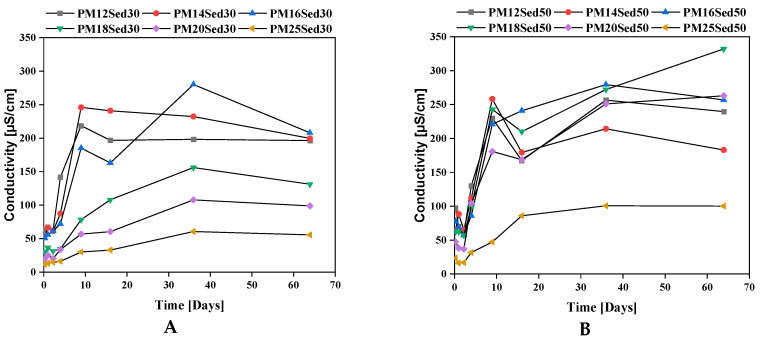
Evolution of conductivity in eluates of polymer mortar including 30% (**A**) and 50% (**B**) of sediments.

It is noted that the conductivity in the first three points is stable except for the polymer mortars with 12% of the quantity of the resin; the second three points of the conductivity increase to the 7th point after it becomes stable (Figure 4). It can also be noted that the conductivity of the mortar with 50% sediment is higher than the mortar with 30% sediment; this discrepancy is related to the amount of sediment, and it is noted that the conductivity decreases when the amount of the resin increases.

Finally, we can note that the conductivity values are relatively low compared to the values measured in the batch leaching test for the sediment and sand. This can be explained by the monolithic nature of samples, which limited the transfer of chemical substances in water. As a matter of fact, different release mechanisms could lead to different release patterns in the dynamic leaching test, namely: (i) solubility, (ii) diffusion from the internal porosity of the matrix to the surface, and (iii) surface wash-off (where substances concentrated at the surface of the monoliths may be released at the first contact with water). Among them, diffusion is clearly the most important in the case of polymer mortar because there is a strong correlation between porosity values and the level of conductivity measured in the sample.

**Figure 5:**
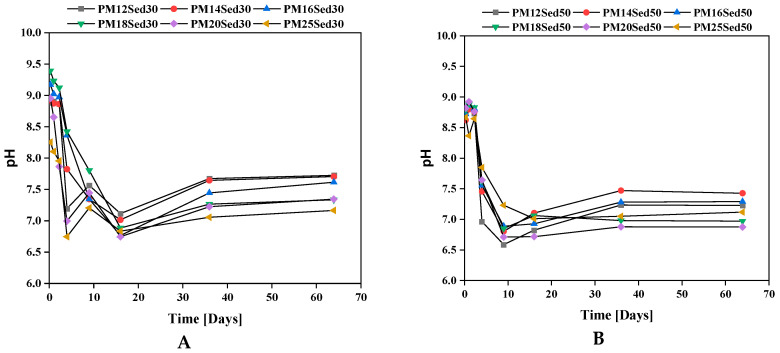
Evolution of pH in eluates of polymer mortar including 30% (**A**) and 50% (**B**) of sediments.

**Figure 6:**
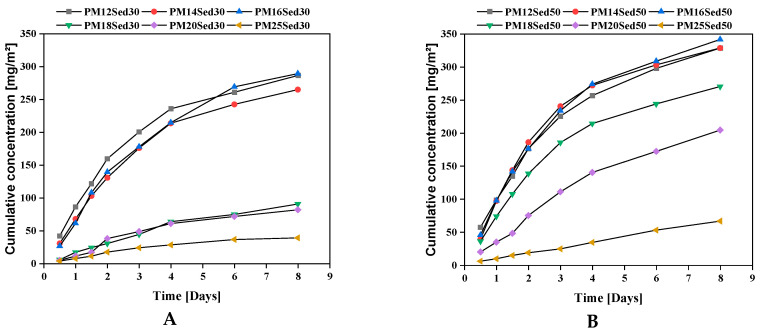
Evolution of cumulative concentration of potassium in leachates from monolithic polymer mortars containing 30% (**A**) and 50% (**B**) of sediments in their matrices.

**Figure 7:**
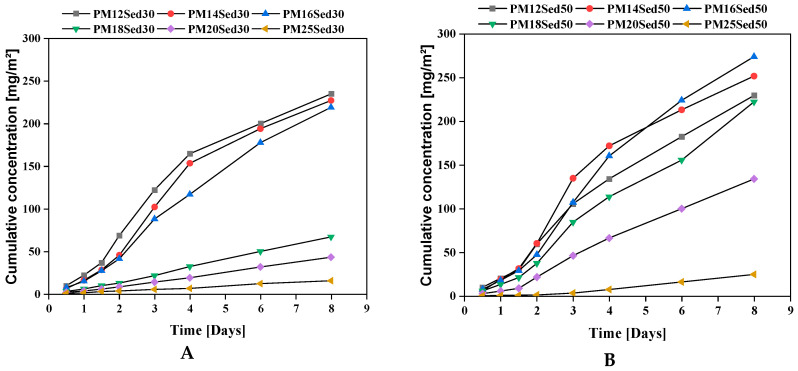
Evolution of cumulative concentration of magnesium in leachates from monolithic polymer mortars containing 30% (**A**) and 50% (**B**) of sediments in their matrices.

This observation is in good agreement with pH evolution (Figure 5) and calcium-leaching mechanisms in the leachates that positively influence the conductivity values. In addition, K and Mg are among the other major elements released by diffusion through polymer mortar matrices, Figure 6 and Figure 7, respectively. The cumulative releases of these major elements are relatively similar and independent of the sediment incorporation rate, suggesting that their concentration is relatively high in the polymer mortar samples. These observations can explain the low discrepancies measured in conductivity in all samples (Figure 4). This is in good agreement with other works on hydraulically bound materials where major elements are diffused in the water and control the ionic background of leachates by diffusion mechanisms [20,21,22,23].

**Figure 8:**
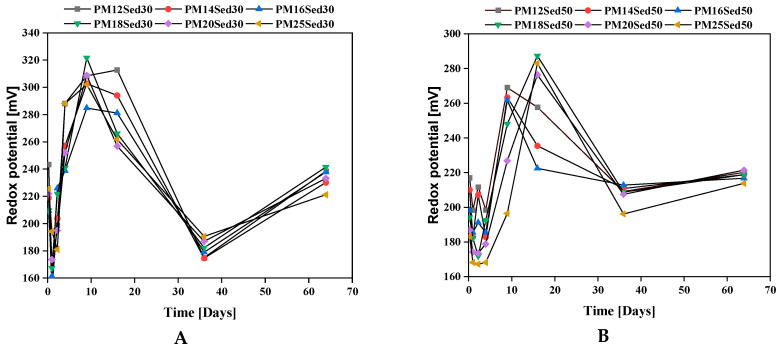
Evolution of redox potential in the leachates from monolithic polymer mortars containing 30% (**A**) and 50% (**B**) of sediments in their matrices.

Redox potential variations were measured in leachates for all formulations and are shown in Figure 8. The values are positive and in the range between 150 and 330 mV for polymer mortars, including 30 and 50%. It means that the medium is oxidized, and there is no redox buffer in the sediment and sand constituents. It can be noted that no difference is measured between all formulations suggesting that this parameter is strongly independent of the chemical composition of polymer mortars.

#### 3.2.2. Sulfates, Chlorides and Fluorides Leaching

**Figure 9:**
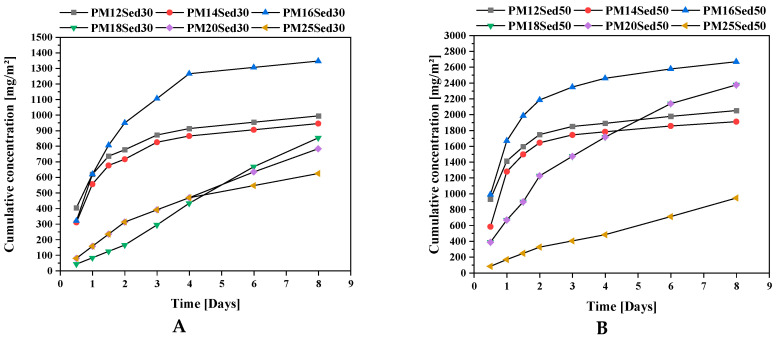
Evolution of cumulative concentration of Sulfates in leachates from monolithic polymer mortars containing 30% (**A**) and 50% (**B**) of sediments in their matrices.

Concentrations in chlorides and fluorides in polymer mortar samples are below the quantification limit, which is consistent with their low concentration in the sediment batch-leaching test and the low porosity of materials. Figure 9 shows the sulfate concentrations in the eight eluates of polymer mortars. When the cumulative sulfate release is considered, fast leaching was noted until 4 days of experiments, where release is dominated by diffusion, and then depletion seems to be reached. After 64 days, the average sulfate cumulative area release is not similar in all formulations. Contrary to the major elements, the sulfate level in leachates is strongly linked to the sediment incorporation rate. Indeed, the increases in sediment incorporation rate tend to increase the diffusion of sulfates. There is no relationship between the resin concentration and sulfates diffusion level in polymer mortars. This suggests that the sulfates are also released by other mechanisms, such as surface leaching or dissolution process from gypsum included in the sediment matrices.

#### 3.2.3. Leaching of Trace Elements

Concentrations of trace metals in polymer mortar leachates were consistently below the limits of quantification for most elements, i.e., As, Cd, Cr, Mo, Ni, Pb, Sb, Se, and Zn, which was in accordance with their low mobility in the sediment and the low porosity of monolithic samples. These results are in good agreement with previous work on asphalt mortar or hydraulically bounded materials [17,18,19]. The system product/water is characterized by two compartments in the exchange flows, i.e., the pore-water in the pores of the product and the leachate compartment. Once the product was brought into contact with water, the system tends towards a new equilibrium state by the transport process (composition of leachate different from that of the pore-water). Different processes take place, including (i) in the porous matrix: dissolution/precipitation processes, chemical reactions (acid/base, complexation, and redox) in the liquid phase, diffusion of soluble chemical species through the pores (from the core to the surface of the product) and transfer to the liquid; (ii) in the leachate: chemical reactions between species, dissolution/precipitation reactions at the product/leachate interface (corrosion of the material surface), transport with the leachate and possibly interaction with a gas phase. The most trace elements present in the sediment, i.e., As, Cd, Cr, Mo, Ni, Pb, Sb, Se and Zn, seem to be strongly influenced by the physical properties of resin that reduce their availability in the leachate.

**Figure 10:**
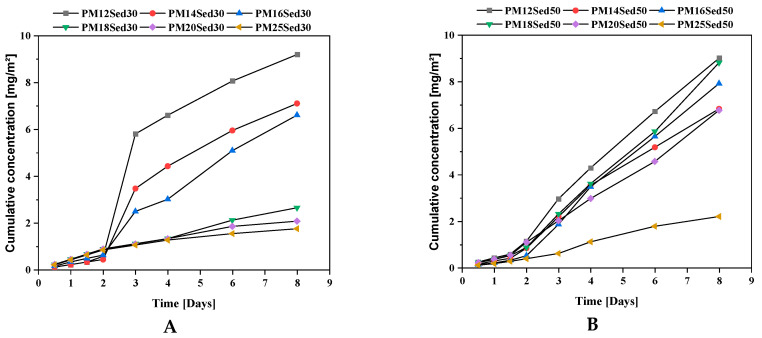
Evolution of the cumulative concentration of Barium in leachates from monolithic polymer mortars containing 30% (**A**) and 50% (**B**) of sediments in their matrices.

**Figure 11:**
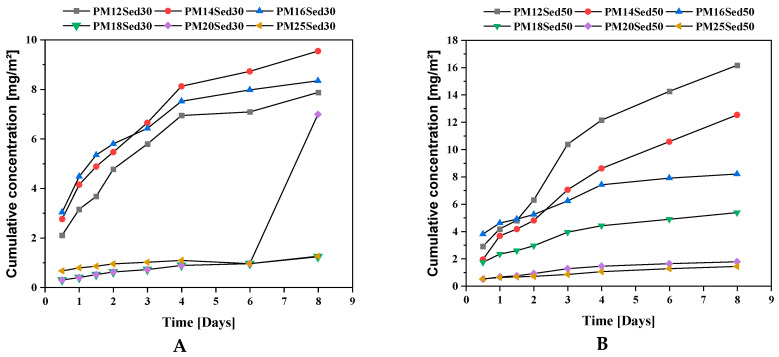
Evolution of cumulative concentration of Cobalt in leachates from monolithic polymer mortars containing 30% (**A**) and 50% (**B**) of sediments in their matrices.

**Figure 12:**
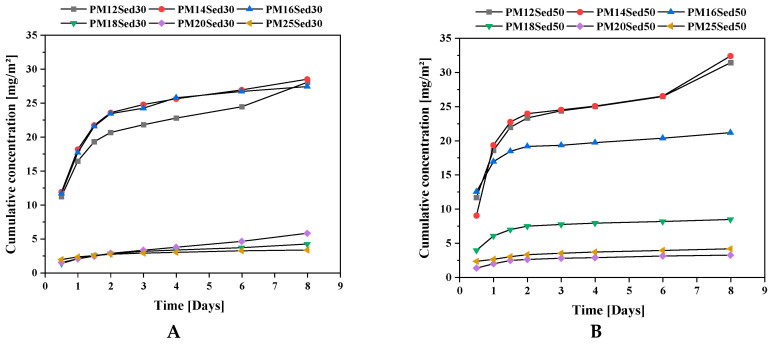
Evolution of the cumulative concentration of Copper in leachates from monolithic polymer mortars containing 30% (**A**) and 50% (**B**) of sediments in their matrices.

In addition, contrary to the cement-based material, no pH modification was observed in the polymer mortar (pH near neutral), suggesting that this treatment process does not change the speciation of metals in the treated sediment, which is why the environmental risk is reduced. Figure 10, Figure 11 and Figure 12 show the evolution of the cumulative concentration of Ba, Co, and Cu, respectively, as a function of time. A linear relationship can be found between the cumulative Ba concentrations and the time, suggesting that this chemical is mainly released by diffusion through polymer matrices.

Sediment concentration plays an important role in the availability of Ba in the leachates with very low concentrations in formulations, including 30% of the sediments and the lowest resin epoxy concentrations. In the formulations, including 50% of the sediments, Ba was detected in all formulations (from the lowest to the highest resin concentration), and a negative relationship can be found between the resin concentration and the level of Ba in leachates. This is clearly linked to the diffusion mechanism that is more significant when the porosity of the polymer mortars increases and, therefore, the resin content decreases. Regarding the relationship between porosity and a polymer binder, the content was detailed in a previous article [13,14]. The behavior of Co is very similar for all mixtures. Indeed, the Co concentration is reduced in formulations including 30% of sediments, and a linear relationship is described between cumulative concentrations and the time for formulations including 50% of sediments. The diffusion process is the main mechanism for the leaching of this element from the matrix.

**Figure 13:**
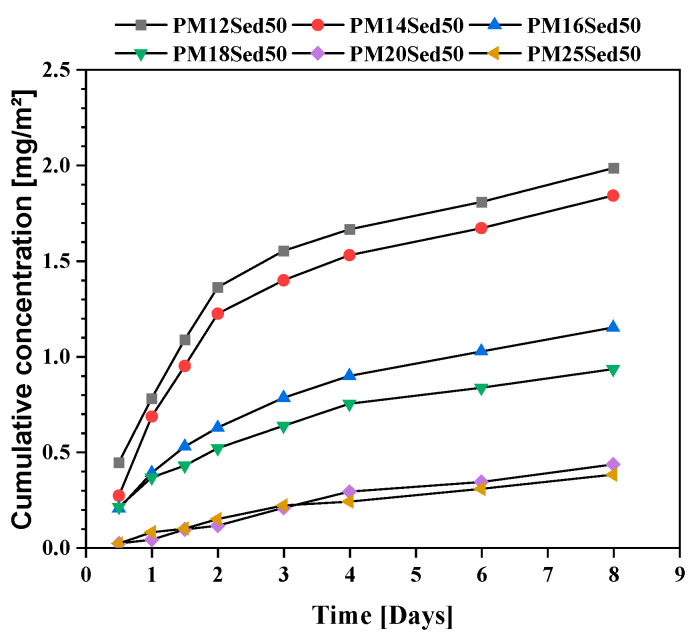
Evolution of cumulative concentration of Vanadium in leachates from monolithic polymer mortars containing 50% of sediments in their matrices.

Figure 13 reports the leaching of V as a function of time for polymer mortars, including 50% of sediments. As observed previously, there is a strong correlation between the resin concentration and the leaching rate measured. The diffusion mechanism observed in the first renewal (until 36 days) is more significant when the resin incorporation rate decreases. This is also linked to the diffusion mechanism that is more significant when the porosity of the polymer mortars decreases with the decrease in polymer resin.

For Ba, Co, Cu, and V, the main mechanism controlling the release at the beginning is the diffusion, and after some renewal, the mechanism changes due to their solid phase speciation and water accessibility to sediment particles entrapped in the polymer matrices. The amount of epoxy binder affects the cumulative heavy metal concentrations. This can be explained by the fact that increasing the porosity tends to increase the water surface contact and water absorption of the polymer mortars. The chemical parameters are strongly influenced by the sediment incorporation rate. Overall, the low leached content of polymer mortar materials is linked to their low hydraulic conductivity and the low polarity of the polymer binder, which prevents water from permeating through the specimens and solubilizes chemical substances. The cumulative releases of heavy metals from polymer mortar are in the same range as cement- and geopolymer-based materials [19,22,23].

#### 3.2.4. Conformity of Construction Products

The results of the standardized release tests (Table 6) can be directly compared to the limit values stated in some countries (e.g., Austria, the Flanders region of Belgium, Denmark, Finland, France, the Netherlands, and soon, also Germany). These limit values are mostly for the granular mineral products in civil engineering works. Some apply to all construction materials (the Netherlands), while others stipulate the limit values for certain materials in specific constructions (Germany, Denmark and Finland). In the Netherlands, the “Soil Quality Decree” sets limits on the values for all stony construction materials, granular or monolithic, and for contaminated soils. Metals and salt contaminants have limited values for their leaching from construction materials. Moreover, organic pollutants have limited values for total content [18]. The Dutch Decree does not separate the products and secondary raw materials. Furthermore, the regulation includes an obligation to remove the material after its service life has ended. The leaching tests results highlight that highly soluble chemicals, such as Ba, Co, Cu, V and sulfates, are systematically detected in all leachates (for some formulations), while some other elements may occur only quickly for the polymer mortars, including the lowest percentage of sediments. The amounts of chemical substances released from all materials comply with the limit values for monolithic construction materials proposed by the Netherlands in their Soil Quality Decree. Such results are, of course, satisfactory from the point of view of the environmental quality of the products because no adverse effect is to be expected for human and environmental health. The strong trapping of the most hazardous substances, such as metallic and metalloid elements within sediment matrices, can be explained by the fact that the use of a polymer is recognized as a treatment process that effectively reduces the environmental availability of contaminated wastes by physical trapping.

## 4. Conclusions

The present study assessed the release of inorganic substances from polymer mortar samples, including dredged sediment as a replacement for a sand fraction—these construction products are typically used for floor coverings in Europe. A diffusion test with the sequential renewal of water was performed in lab conditions for different resin epoxy concentrations and sediment incorporation rates. This test was conducted according to specifications described in the NF EN 15863 standard. It can be concluded that the release of soluble substances is very limited in these hydrodynamic conditions. This is particularly true for high epoxy-resin concentration leachates where no trace element, except for small quantities of barium, cobalt, copper, and vanadium, are quantified. Their leaching is mainly controlled by diffusion in the first renewal steps, and then the depletion is observed for all formulations. This is because of the low hydraulic conductivity (low porosity and water absorption) and the low polarity of the polymer binder of these specimens. The percentage of sediment included in the polymer mortars plays important on the leaching of sulfates and major elements (Ca, Fe, K and Mg) by improving their diffusion/dissolution/surface leaching at the highest incorporation rate (50%).

No adverse effect is to be expected in terms of environmental health from the leachates of these polymer mortars because all the measured parameters were below the Soil Quality Decree limits established in the Netherlands. These data about the environmental performances of road construction materials, including dredged sediments, are the first to be published and may serve as a basis for identifying the amounts of hazardous substances that such construction products may release in water. They are of great interest to potential users of secondary raw materials and are required for the CE-marking procedure of construction products. Finally, it seems that pollution from runoff water is more likely to be related to the chemical products applied to the polymer mortars than to the materials themselves. However, an extrapolation of these results to the field conditions must be done with great caution due to the very different hydrodynamic conditions (L/A ratio, leachate renewal, etc.) and evolution of the construction materials under climatic changes (degradation, oxidation of polymer matrix) may be observed during floor covering use. Moreover, further experiments should be performed on a larger panel of dredged sediments to gain a better knowledge of their potential to release substances into the water.

## Figures and Tables

**Table 1:** Main characteristics of the used epoxy resin.

Proportions of the Mixture	1 Part of Hardener: 2 Parts of Basic Solution (by Weight)
Density	1.1 g/cm^3^
Hardness Shore D	70–75
Hardness of the core	70–75 N/mm^2^ at 14 days
Heat resistance	+40 °C to +45 °C
Operating temperature	+10 °C to +30 °C
Viscosity	1000–1200 mPa·s

**Table 2:** Physical characterization of the sediment.

Characteristics	Standards	Sediment
Density (Kg/m^3^)	NF EN 1097-7	2610
Methylene blue value (g/100 g of dry matter)	NF P 94-068	0.53
Organic matter content (%) at 450 °C	XP P94-047	4.2
BET Surface (m^2^/g)	NF EN ISO18757	11.01

**Table 3:** Chemical composition of raw sediment according to X-ray fluorescence analysis.

Elements (%)	O	Na	Mg	Al	Si	P	S	Cl	K	Ca	Ti	Fe
Content	48.5	0.4	0.9	6.7	24.8	0.5	0.4	Traces	1.8	11.8	0.5	3.6

**Table 4:** Main mechanical and physical characteristics.

Mix	30% Sediment and 70% Sand						50% Sediment and 50% Sand					
Resin (%)	12	14	16	18	20	25	12	14	16	18	20	25
Porosity (%)	26.41	25.06	14.29	3.95	7.27	6.51	34.97	38.68	24.04	19.21	13.99	11.03
Water absorption (%)	1.20 × 10^−1^	1.11 × 10^−1^	7.98 × 10^−2^	1.60 × 10^−2^	2.81 × 10^−3^	1.22 × 10^−3^	2.57 × 10^−1^	2.22 × 10^−1^	1.52 × 10^−1^	6.85 × 10^−2^	3.21 × 10^−2^	2.27 × 10^−3^
Density g/cm^3^	1556	1600	1600	1830	1890	1960	1400	1370	1450	1600	1730	1830

**Table 5:** Leaching test results of raw sediments and sand according French regulation [20].

Parameters	Sediment	Sand	Inert Waste Threshold	Non-Hazardous Waste Threshold
As	<0.1	<0.1	0.5	2
Ba	3	0.03	20	100
Cd	<0.01	<0.01	0.04	1
Cr	0.02	<0.01	0.5	10
Cu	0.6	<0.02	2	50
Mo	0.1	<0.05	0.5	10
Ni	0.1	<0.04	0.4	10
Pb	0.1	<0.02	0.5	10
Sb	0.11	<0.05	0.06	0.7
Se	0.07	<0.07	0.1	0.5
Zn	1.0	<0.03	4	50
chlorides	36	<10	800	15,000
fluorides	20	<5	10	150
sulfates	270	<10	1000	20,000
soluble fraction	2837	358	4000	60,000
pH	8.09	8.98	-	>6
Conductivity (µS/cm)	264	27.75	-	-

**Table 6:** Comparison of the cumulative release of Ba, Co, Cu, V and sulfates (mg/m^2^) at 64 days in the monolith leaching test with regulatory leaching limits established in the Netherlands within the Soil Quality Decree (SQD).

Formulations		30% Sediment and 70% Sand						50% Sediment and 50% Sand						SQD Leaching Limits
Resin Rate (%)		12	14	16	18	20	25	12	14	16	18	20	25	
Parameters	Ba	9.19	7.10	7.10	2.64	2.07–	1.75	9.01	6.83	7.92	8.82	6.77	2.2	1500
	Co	7.87	9.54	8.35	1.5	1.8	1.7	16.16	12.53	8.2	5.37	1.77	1.43	60
	Cu	28.0	28.5	27.4	4.2	11.2	4.2	31.4	32.4	21.1	8.43	3.22	4.14	98
	V	-	-	-	-	-	-	1.99	1.84	1.15	0.93	0.43	0.38	320
	Sulfate	994	954	1346	853	782.81–	624.02–	2050	1911	2669	2569	2376	944.28–	165,000

## Data Availability

https://www.sedilab.com/portfolio-item/sediplast/ (accessed on 4 December 2022).
